# Progress in Fevers

**Published:** 1902-02-15

**Authors:** 


### Progress in Fevers.

**Small-pox.**—The last great epidemic of small-pox—European in extent—occurred 30 years ago, in 1871. As in that epidemic, London appears to have received the present disease from the Continent.[1] Small-pox has prevailed in Paris since June, 1900, and up to September, 1901, had caused 543 deaths.[2] Although, owing to improved isolation, a repetition of the terrible mortality of the earlier period need not be feared – 7,912 persons died then in London alone—a new source of danger arises from the fact that many children have, of recent years, escaped vaccination. Published figures would show that of the children born during the last few years in London, one-fourth to one-third have "not been accounted for" as regards vaccination. The number of persons over 10 years of age who have not been re-vaccinated must also be very large. The lack of union in the working of the various authorities, the Health and Board School authorities and the public vaccinator, greatly hinders prompt vaccination when infection has arisen or is likely to arise. The Local Government Board has issued circulars to the Metropolitan borough councils and boards of guardians urging prompt action in the matter of vaccination, re-vaccination, and isolation, and urging also, among other things, that "contacts" should be kept under observation for not less than 14 days. Resolutions aimed to secure these measures were passed in September by the St. Pancras Borough Council, in spite of the anti-vaccination proclivities of Mr. Bernard Shaw,[4] and also a resolution making chicken-pox notifiable for six months. The rarity of small-pox of late years and the difficulty of distinguishing mild or modified cases from chicken-pox is also a source of danger. To overcome this last difficulty[5] Mr. Wynter Blyth suggests that the Metropolitan Asylums Board should publish a set of carefully prepared life-size lithographs of selected cases specially difficult to diagnose, for the use of medical men who have had no opportunity of becoming acquainted with the disease. Referring to the old plea of the anti-vaccinationists that the decline in the prevalence and severity of small-pox is due to improved sanitation, one writer[6] reverts to the last epidemic in Gloucester in 1896-7.

Although Gloucester is not a particularly insanitary place, it can hardly be called a sanitary model. Three main collectors discharge their crude sewage into a narrow branch of the Severn. Near to this outlet is situated the oldest, poorest, and most overcrowded part of the city. It was not here, however, but in the newer, better portion of the town, consisting chiefly of villa residences, that the great severity of the disease was experienced. The Medical Officer of Health, Dr. Campbell, showed in his report that cases were extremely rare in properly vaccinated children under 10 years, and mild if they did occur; while in the unvaccinated the incidence of the disease and the mortality were very high. It was found that quite rabid anti-vaccinationists promptly sought vaccination when danger pressed! Spalding, of Chicago,[7] refers to the epidemic of mild, irregular small-pox recently described by Montizambert as occurring in Canada, and variously called chicken-pox, impetigo contagiosa, cedar itch, etc. In Chicago men in the pustular stage have been found working in the factories and business houses. Like Montizambert, Spalding considers the disease true though mild small-pox, for hæmorrhagic and confluent cases occasionally occur, and "the disease follows the rule of small-pox in attacking exclusively those not protected [efficiently] by vaccination." Spalding[8] further points out that in all the cases, however mild, there is general conformity to the type of an ordinary case, some prodromal symptoms are always present and subside when the rash appears. This should be sought for on the vascular parts. Umbilication in mild cases may be absent; but the lesion, involving the true skin, is always deeply placed, and feels "shotty." An irregular mild case, while it confers immunity on the individual, may give rise to typical confluent or hæmorrhagic small-pox in others. Coffman[9] also calls attention to the same mild type of disease prevalent in several American cities and often mistaken for impetigo contagiosa, from which, as from chicken-pox, it is distinguishable by the initial symptoms commencing 48 hours before the rash. J. H. Crocker[10] records three cases of small-pox occurring at Hounslow. One, a woman aged 39, noticed spots one day before delivery. Death occurred eight days after delivery. There was no evidence of peritonitis. The rash was of a bad confluent type. Her infant son was vaccinated four days after birth with lymph which "took well" in 20 other cases, and again subsequently. No result followed. Eight days after birth he became feverish; two days later the rash, which became confluent, appeared, and death ensued ten days later. Since the rash appeared ten days after birth, the probability is that the disease was contracted *in utero*.

The third case, a man aged 25, well vaccinated in infancy, developed papules after one day's *malaise*, only one of which became pustular and umbilicated. Vaccination, performed after the rash appeared, "took" in three places out of four, although the course of the vaccinia was much delayed. Notwithstanding the vaccination result, the case was regarded as varioloid rather than chicken-pox. Recently, since the demand for lymph has so increased, complaints[11] have been made of the inertness of some of that supplied commercially. A question of such vital importance as the efficiency of the lymph supplied demands sifting to the very bottom. It has been asked if it would not be possible for the Local Government Board to supply not only the public vaccinators, as at present, but private practitioners also, with lymph from their laboratories. When, on re-vaccination, a much-modified result follows, in a person not efficiently vaccinated for a long time, a second or even third attempt should be made before protection is assumed. In the region of pathology Roger and Veil[12] describe and figure the corpuscular elements already noted by other observers in the pus of variolous pustules and variously considered nuclear *débris* or parasites. They are minute, rounded bodies, staining more deeply than the cell nuclei with Loeffler's blue, found in the pustules, but also in the papules and blood. They were also abundant in the amniotic fluid of two pregnant women who died of small-pox, and showed some motility in this medium. No leucocytes were present in the fluid, and the bodies appeared to be surrounded by a thin film of protoplasm. Rabbits injected with variolous pus died of a septicæmia in about three weeks. Cultivations of their blood showed the bodies, which retained their characteristics through 18 sub-cultures. Roger and Veil conclude the corpuscles are not nuclear *débris*, nor of vegetable origin, but that they are probably protozoa, and that they have something to do with the causation of small-pox.

[1] Lancet, Sept. 21, p. 796. [2] Brit. Med. Jour., Sept. 28. [3] Ibid. [4] Lancet, Oct. 19; Brit. Med. Jour., Oct. 5 and Oct. 26. [5] Brit. Med. Jour., Sept. 14, p. 727. [6] Lancet, Aug. 24. [7] Brit. Med. Jour., Sept. 21, p. 821. [8] Med. Rec., Sept. 28. [9] Med. Rec., Sept. 21. [10] Lancet, Nov. 2. [11] Brit. Med. Jour., Nov. 16. [12] Practitioner, Sept.

